# Supplementary material for: Simulation of transient rolling resistance of bicycle tyres at various ambient temperatures
Source: PLoS One. 2024 Jun 27;19(6):e0302821. doi: 10.1371/journal.pone.0302821 (PMC11210805; doi:10.1371/journal.pone.0302821)
Supplement: Attachment — Submitted filename: plosone_review_rr.docx [file pone.0302821.s002.docx]

Review for manuscript: “Simulation of non-stationary rolling resistance of bicycle tyres at various ambient temperatures”

This study examines the effect of ambient temperature on bicycle rolling resistance. It is suggested that more energy demand due to higher rolling resistance at low ambient temperatures (0 to -30C) affects the commuters’ choice to use bicycles as the preferred method of commuting. Different components of total driving loss are explained, and total rolling resistance is derived. Rolling resistance and tyre temperature are then plotted as a function of ambient temperature. The calculations are then repeated for an electrically assisted bicycle and similar trends are observed in rolling resistance and tyre temperature. It is also suggested that lower battery capacity in low ambient temperatures must be considered in battery design.

The referred paper which studied the stationary model of rolling resistance is not accessible to be able to compare the two studies and comment about the level of novelty of the work and the implementation of non-stationary assumption in this model is not clear.

This manuscript could be published in PLOS ONE journal subject to addressing the following comments.

Grammer:

1- In the third line of the abstract the word “commuter” is repeated twice, and the structure of the sentence should be revised.
2- Line 74, “better understanding rolling resistance”, this sentence’s grammar must be revised.
3- Line 119: The word “road” is repeated, you may edit this sentence.
4- Line 293: You may change this sentence to: “…ambient and tyre temperatures.”

Technical comments:

1- In the last line of the abstract, please specify a range for very low ambient temperatures.
2- Line 41: Is the gain factor a parameter? If so , you may define it.
3- Line 44: please explain what you mean by range anxiety and whether it was meant to be pedelec or electric cars.
4- Line 66: Which factor do you think has the most significant effect on the rolling resistance among the factors mentioned? Does ambient temperature have the highest impact?
5- Rolling resistance force is mentioned as F_r_ in equation (1) but labeled as F_rr_ in Figure 1
6- If this is Newton’s second law, should not F_total_ be equal to zero? Where was this derivation applied to create the results?
7- Line 101: You may replace weight with mass.
8- Please add units for all the parameters and variables in the equations.
9- F_c_ in figure 2 is not defined.
10- Small v is used in equation (3) and capital V in equation (6) for velocity. You may use the same notation for the variables.
11- Line 155: I assume it was meant to be “rolling resistance coefficient”.
12- Define b_1_ and b_2_ in equation (12).
13- Equation (17) is not clear, h_free_  which is mentioned in the text is not used in this equation and h_natural_ is not defined.
14- Where is the non-stationary assumption implemented in the derivations compared to the referred steady-state study?
15- Unit of the vertical axis in figure 6 is missing.
16- Line 281: The sentence is incomplete.
